# Synthesis and Characterization of Quadrupolar-Hydrogen-Bonded Polymeric Ionic Liquids for Potential Self-Healing Electrolytes

**DOI:** 10.3390/polym14194090

**Published:** 2022-09-29

**Authors:** Chenming Li, Rajesh Bhandary, Anja Marinow, Dmitrii Ivanov, Mengxue Du, René Androsch, Wolfgang H. Binder

**Affiliations:** 1 Macromolecular Chemistry, Institute of Chemistry, Faculty of Natural Science II, Martin Luther University Halle-Wittenberg, Von-Danckelmann-Platz 4, 06120 Halle (Saale), Germany; 2 Interdisciplinary Center for Transfer-Oriented Research in Natural Sciences, Martin Luther University Halle-Wittenberg, 06099 Halle (Saale), Germany

**Keywords:** RAFT polymerization, hydrogen bonds, polymeric ionic liquids

## Abstract

Within the era of battery technology, the urgent demand for improved and safer electrolytes is immanent. In this work, novel electrolytes, based on pyrrolidinium-bistrifluoromethanesulfonyl-imide polymeric ionic liquids (POILs), equipped with quadrupolar hydrogen-bonding moieties of ureido-pyrimidinone (UPy) to mediate self-healing properties were synthesized. Reversible addition–fragmentation chain-transfer (RAFT) polymerization was employed using S,S-dibenzyl trithiocarbonate as the chain transfer agent to produce precise POILs with a defined amount of UPy and POIL-moieties. Kinetic studies revealed an excellent control over molecular weight and polydispersity in all polymerizations, with a preferable incorporation of UPy monomers in the copolymerizations together with the ionic monomers. Thermogravimetric analysis proved an excellent thermal stability of the polymeric ionic liquids up to 360 °C. By combining the results from differential scanning calorimetry (DSC), broadband dielectric spectroscopy (BDS), and rheology, a decoupled conductivity of the POILs from glass transition was revealed. While the molecular weight was found to exert the main influence on ionic conductivity, the ultimate strength and the self-healing efficiency (of up to 88%) were also affected, as quantified by tensile tests for both pristine and self-healed samples, evidencing a rational design of self-healing electrolytes bearing both hydrogen bonding moieties and low-molecular-weight polymeric ionic liquids.

## 1. Introduction

Lithium-ion batteries (LIBs) as an alternative power source to traditional petrochemical energy have attracted researchers’ interest owing to their excellent power density, reusability, and processability [1]. However in conventional liquid electrolytes, LIBs generally suffer drawbacks from lithium dendrites [2,3], leakage and flammability of the liquid electrolytes and the thus resulting poor thermal stability [4]. To solve such deficiencies in current electrolyte systems, polymeric ionic liquids (POILs), where ionic moieties are tethered onto a polymeric backbone, represent potential candidates as polymer electrolytes due to their good conductivity, thermal and mechanical stability, as well as their plasticity [5,6]. For next-generation electrolytes with battery applications, not only high ionic conductivity and good mechanical properties are required [7,8], but also properties such as self-healing can contribute to extending the lifespan of a polymer electrolyte, and thus of the battery as a whole. In the field of supramolecular materials, hydrogen bonds (HBs) play an important role because HBs can provide interchain forces, leading to material properties such as improved mechanical strength [9], shape memory behavior [10], stimulus responsiveness [11], and self-healing [12,13]. Thus in the field of polymer electrolyte chemistry, researchers are exploiting such HBs in POILs to exploit their generally proposed weaker strength and thus to embed self-healing ability into the materials [14,15]. Among the diverse HB synthons, ureido-pyrimidinone (UPy) is widely applied to polymer matrices [16,17,18,19] owing to its facile synthesis and strong dimerization-abilities (with an association constant, *K*_asssn._ ≥ 10^6^ M^−1^ in CDCl_3_) [20] via its quadrupolar HBs. There have been several approaches to introducing UPy into POIL to explore its self-healing property together with the conductive nature of POILs [15,17,18,19], which represents an advantageous strategy to design new-generation functional electrolytes.

While many POILs have been reported predominantly with polyimidazolium ions [17,21] or other POILs [18,22,23] as self-healing electrolytes, poly-pyrrolidinium-based self-healing electrolytes have been scarcely reported, despite the fact that pyrrolidinium-based ionic liquids generally have a higher electrochemical stability [24]. Moreover, in many publications POILs were synthesized via free radical polymerization, which offers no control over the molecular weight of POILs, thus yielding polymers with a broad polydispersity and a poor reproducibility, which significantly influences the final conductivity of the obtained polymer electrolytes [25,26,27,28]. While UPy was widely exploited in polymers synthesized via RAFT polymerization [29,30,31], only a few [32] RAFT kinetics studies regarding an acrylate-based UPy monomer were reported, let alone studies involving its copolymerization with ionic monomers.

Motivated by these findings, we herein report the synthesis and characterization of pyrrolidinium-bistrifluoromethanesulfonyl-imide-based POILs (**POIL-x**, with x being the entry number as provided in Table 1) and also the copolymers (**CPILU-y**, with y being the entry number) with an acrylate-based UPy monomer via RAFT polymerization. Kinetic studies were carried out firstly via the RAFT homopolymerization of the ionic monomer **ILA** in DMF, and subsequently the copolymerization kinetics with the acrylate-based UPy monomer **UPyA**. Homopolymers and copolymers with various molecular weights were prepared, and their thermal, electrical, rheological, and self-healing properties were discussed. Thermogravimetric analysis (TGA) revealed an extraordinarily high thermal stability of the homopolymers **POIL-x** and the copolymers **CPILU-y**, while differential scanning calorimetry (DSC) was employed to study the thermal transition of the homo- and co-polymers. Together with conductivity obtained via broadband dielectric spectroscopy (BDS) and zero shear viscosity determined by rheology, the relationship between conductivity and viscosity was explored. By combination with DSC data, an insight into the decoupled conductivity properties of both **POIL-x** and **CPILU-y** was revealed. To elucidate the self-healing ability, two copolymers with different molecular weights but identical UPy content were subjected to tensile tests, allowing the future rational design of self-healing electrolytes via HBs and with low molecular weight.

## 2. Materials and Methods

Acryloyl chloride 96%, 2-cyanobutanyl-2-yl 3,5-dimethyl-1H-pyrazole-1-carbodithioate, 95% (PCDT), and calcium hydride coarse powder 92% were purchased from ABCR (Karlsruhe, Germany); 3-chloro-1-propanol 98%, 1-methyl-pyrrolidine 98%, benzyl chloride 99%, carbon disulfide 99.9%, and α,α′-azoisobutyronitrile (AIBN) were purchased from Sigma-Aldrich (Taufkirchen, Germany); triethylamine 99% and molecular sieve 3Å 1–2 mm bead were purchased from Alfa Aesar (Kandel, Germany); 2-isocyanatoethyl acrylate 98% and 2-amino-4-hydroxy-6-methylpyrimidine (6-methylisocytosine) 98% were purchased from TCI (Eschborn, Germany); lithium bis(trifluoromethanesulfonyl)imide 99% was purchased from IoLiTech (Heilbronn, Germany); and potassium carbonate was purchased from Bernd Kraft (Oberhausen, Germany). Molecular sieve 3Å was activated in a vacuum oven at 200 °C for one week before use. 1-Methyl-pyrrolidine was refluxed and distilled with calcium hydride for 48 h to dry and stored with molecular sieve 3Å in a glovebox filled with nitrogen (O_2_: 0.1 ppm, H_2_O: 0.1 ppm). All the other chemicals were used as received unless otherwise stated. All other chemicals which are not described here can be found in the Supplementary Material Section S1.1.

Thermogravimetric analysis (TGA) was performed using a Netzsch Tarsus TG 209 F3 (NETZSCH-Gerätebau GmbH, Selb, Germany). Approximately 10 mg of samples was placed in aluminum oxide crucibles and measured from room temperature to 600 °C at 10 K·min^−1^ under nitrogen with a flow rate of 20 mL min^−1^. The data analysis was performed via the NETSCH Proteus-Thermal Analysis (Germany, version 5.2.1) software.

Differential scanning calorimetry (DSC) measurements were performed on a calibrated heat-flux DSC (Mettler-Toledo, Greifensee, Switzerland) equipped with a FRS5 sensor, connected to a TC100 Intracooler (Huber, Offenbach, Germany). 5–10 mg of samples were placed in aluminum sealed crucibles for measurements. The thermal history was cancelled by heating the samples from 25 °C to 120 °C at 10 K·min^−1^, followed by isothermal annealing for 20 min, re-cooling to −60 °C at 5 K·min^−1^, and thermostatting at −60 °C for another 20 min. The final DSC curves were then recorded from −60 °C to 120 °C at 5 K·min^−1^.

Preparations of samples applied in this work with specific geometry (bar or disc) were obtained via hot-vacuum press by a VCM Essential molding machine (MeltPrep GmbH, Graz, Austria). First, the raw polymers were dried in an oven at 80 °C at 5 mbar for 24 h and then dried thoroughly by an ultrahigh vacuum pump at 80 °C for 24 h to eliminate water traces. Second, the polymers were hot-vacuum-pressed into a bar or disc at 70 °C and the so prepared specimens were kept in a desiccator with phosphorous oxide before the measurements started. For disc samples with specific geometries, a punching tool was used. For the self-healing samples, the pristine samples were cut vertically at the middle of the long edge and brought back tightly before being put in an oven pre-conditioned at the temperature of interest and annealed for a certain time. After self-healing, the samples were cooled to room temperature in a desiccator containing phosphorous oxide before measurements were conducted.

Broadband dielectric spectroscopy (BDS) was performed on an Alpha analyzer dielectric spectrometer (Novocontrol Technologies GmbH & Co. KG, Hundsangen, Germany). Two different sample cells were used depending on the sample texture. For the gel samples, a cell consisting of two brass electrodes with a sample size of 20 mm diameter and a thickness of 0.25 mm was used. Gel samples were carefully inserted into the hollow space between the electrodes inside the sample holder, with air bubble formation avoided. For self-standing samples, the samples were firstly hot-vacuum-pressed into a disc of 20 mm in diameter and 0.2–0.8 mm in thickness and subsequently sandwiched between two brass electrodes for the dielectric measurement. The measurements were performed inside a cryostat with a constant flow of dry nitrogen gas during the measurements to avoid moisture. Measurements were performed in a frequency range from 1 Hz to 10^6^ Hz. Values of the DC conductivity were extracted from the DC plateau of the log *σ* vs. log frequency plots.

Rheology measurements were performed on an MCR 101 DSO rheometer (Anton Paar Germany GmbH, Ostfildern-Scharnhausen, Germany) using a parallel plate–plate geometry (plate diameter: 8 mm). All polymers were dried under high vacuum at 80 °C for 24 h before the rheology measurement. While the gel samples were directly smeared on the lower plate of the sample holder, the solid samples were hot-vacuum-pressed into films at 70 °C, cut into discs of 8 mm with a punching tool, and subject to the measurement. The sample temperature was regulated by thermoelectric cooling/heating in a Peltier chamber under a dry nitrogen atmosphere. At each temperature the sample was equilibrated for 20 min before measurement was initiated. All measurements were performed in the dynamic mode and repeated twice to ensure precise viscosity values. Data analysis was performed via Rheo Compass^TM^ (Austria, version V1.30.1064).

Tensile tests were performed on an INSTRON 5900 Series (INSTRON Deutschland GmbH, Darmstadt, Germany) tensile testing machine at room temperature at a strain rate of 20 mm·min^−1^. Data analysis was performed via Bluehill Universal (Germany, version 4.08).

All methods which are not mentioned here can be found in the Supplementary Material Section S1.1.

## 3. Results and Discussion

### 3.1. Synthesis of Monomers and Polymers

**Scheme 1:**
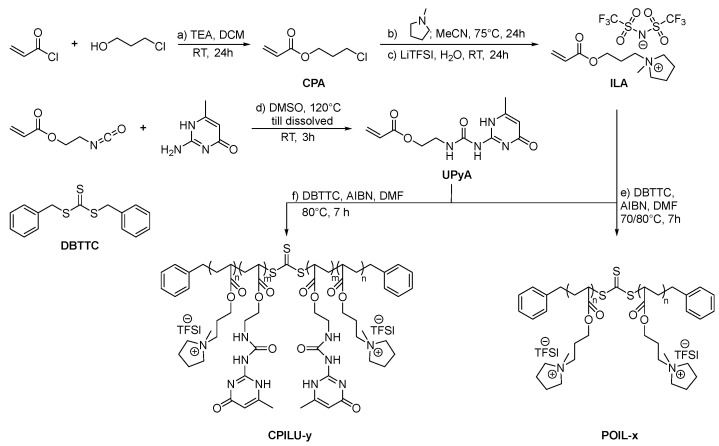
Synthetic route towards the **POIL-x** and **CPILU-y** (bearing the IL and the self-healing UPy moieties): (**a**) precursor **CPA**: acryloyl chloride with chloropropanol and triethylamine (TEA) at room temperature for 24 h; (**b**) ionic monomer **ILA**: **CPA** with 1-methyl pyrrolidine in MeCN at 75 °C for 24 h; (**c**) with LiTFSI in H_2_O at room temperature for 24 h; (**d**) HBs monomer **UPyA**: 6-methylisocytosine in DMSO at 120 °C, followed by isocyanatoethyl acrylate and stirring at room temperature for 3 h; (**e**) homopolymers **POIL-x**: DBTTC and AIBN in DMF at 70/80 °C for 7 h; (**f**) copolymers **CPILU-y**: DBTTC and AIBN in DMF at 80 °C.

To synthesize the ionic monomer **ILA**, firstly acryloyl chloride was reacted with chloropropanol in excess with triethylamine as the organic base in DCM to obtain chloropropyl acrylate (**CPA**) as a precursor. The precursor **CPA** was then used to quaternize 1-methyl pyrrolidine in MeCN to obtain pyrrolidinium-based acrylate with chloride as the counterion. Ion exchange was performed in water using an excessive amount of LiTFSI to afford the final ionic acrylate-base monomer **ILA**, with the pyrrolidinium-ion representing the cationic moiety and bistrifluoromethanesulfonyl imide (TFSI) constituting the anion (see Scheme 1). To link the UPy moiety to a polymerizable acrylate, 6-methylisocytosine was firstly dissolved in hot DMSO, whereafter an excessive amount of isocyanatoethyl acrylate was added, followed by immediate cooling in a water bath to prevent self-polymerization. After stirring for 3 h at room temperature and subsequent purification, the finely powdered UPy monomer **UPyA** was obtained (for synthetic details, see Supplementary Material Section S1.2).

**Figure 1:**
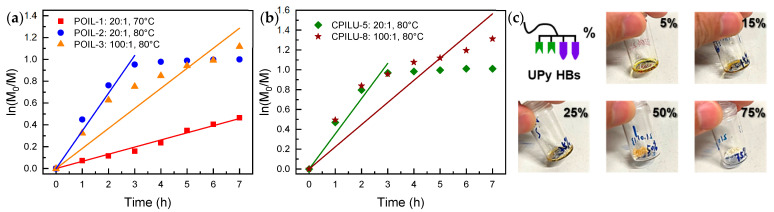
Kinetic plots of RAFT (**a**) homopolymerizations of **ILA** under the conditions: [**ILA**]/[DBTTC] = 20:1 at 70 °C (red square), [**ILA**]/[DBTTC] = 20:1 at 80 °C (blue circle), and [**ILA**]/[DBTTC] = 100:1 at 80 °C (orange triangle); (**b**) copolymerizations of **ILA** with **UPyA** under the conditions: [**ILA** + **UPyA**]/[DBTTC] = 20:1 at 80 °C (green rhombus) and [**ILA** + **UPyA**]/[DBTTC] = 100:1 at 80 °C (brown star); and (**c**) photos of the copolymers **CPILU-y** with various contents of UPy moieties.

In this work, S,S-dibenzyl trithiocarbonate (DBTTC) was used as a chain transfer agent (CTA) for the RAFT polymerization owing to its excellent control over acrylate monomers [33] and ionic monomers [34]. To make full use of the potential of DBTTC and reach the maximum chain end fidelity, the ratio between DBTTC and AIBN was kept at 1:0.1 in all entries. Thanks to the nature of RAFT polymerization, by manipulation of the **ILA** to DBTTC ratio, the molecular weight of the final polymers can be adjusted, which enables precise studies on the influence of molecular weight on the final properties of the homopolymer **POIL-x** and the copolymers **CPILU-y**. RAFT polymerizations were carried out in DMF because it has been reported as an outstanding solvent for the polymerization of ionic liquids [35]. To obtain **POIL-x** with various molecular weights, the kinetics of RAFT homopolymerization of the **ILA** was investigated using DBTTC and AIBN as the initiator. The polymerization conditions, the monomer conversion into the final polymer, the calculated theoretical and practical number-averaged molecular weight via ^1^H NMR, as well as polydispersities determined by gel permeation chromatography (GPC) can be found in Table 1. As shown in Figure 1a), polymerization using [**ILA**]/[DBTTC] = 20:1 at 70 °C (red square) showed first-order kinetics within the investigated time (7 h), while increasing the reaction temperature to 80 °C (blue circle) with the same monomer to CTA ratio drastically boosted the reaction rate, and the polymerization reached its maximum conversion within 3 h. As represented by the orange triangles in Figure 1a, when the [**ILA**]/[DBTTC] ratio was increased to 100:1, the polymerization at 80 °C showed pseudo-first-order reaction kinetics, and after a 7-hour reaction, it reached a final conversion of 67%. As shown in Table 1, when the [**ILA**]/[DBTTC] ratio increased from 20: to 100:1, and subsequently to 200:1, the conversion also rose from 62% to 67%, and finally to 79%. Control over the molecular weight was also improved as the ratio increased, as evidenced by the decreasing deviation between the theoretical and experimental molecular weights calculated via ^1^H NMR (for calculation details and NMR spectra, see Supplementary Material Sections S1.2 and S4). In addition to the moderate control over molecular weight, the control over polydispersity decreased as the [**ILA**]/[DBTTC] ratio increased but still remained within the moderate range (PDI ≤ 1.4).

Although GPC is a well-established method to characterize the molecular weight of many neutral polymers, the determination of the molecular weight of POILs via GPC remains challenging due to their unique hydrodynamic radius and interaction with the GPC column, as previously reported [36,37,38]. In 2013, He [38] reported a strategy to characterize the molecular weight of imidazolium-based POILs with various (counter)-anions by synthesizing a set of poly(4-vinylbenzyl chloride) with precise molecular weights via RAFT polymerization, followed by quaternization and ion exchange of the polymer precursors to obtain the final PS-based polybutylimidazolium with TFSI as the counterion. These polyimidazoliums were then used for calibration in a DMF GPC with LiTFSI as the additive to reduce the interaction of POILs with the GPC column. The molecular weights detected by this GPC were compared with those obtained via ^1^H NMR, proving the preciseness of this strategy. Therefore we also prepared our acrylate-based polypyrrolidinium following this strategy (for synthesis details, see Supplementary Material Section S1.3). However, during the quaternization where 1-methylpyrrolidine was involved, side reactions took place, and the final POILs showed imperfect curves as detected by the GPC in DMF. This could be attributed to the hydrolysis of the ester moieties of the polymer [39,40] and the RAFT-active chain end [41] due to the presence of 1-methyl pyrrolidine (which is a base), which deteriorated the overall polydispersity and further impaired our GPC characterization with POIL calibration. Therefore, only the conventional PS standard calibration together with the 0.1 M LiTFSI as an additive was used in DMF GPC to establish the polydispersity (for GPC curves and data, see Supplementary Section S2).

To introduce the HB-moiety UPy into the POILs and also to study the RAFT copolymerization kinetics of the ionic monomer **ILA** with the quadrupolar hydrogen-bonded monomer **UPyA**, random copolymerizations of the two monomers were investigated. The copolymerization conditions, the monomer conversion into the final polymer, the initial molar fraction of **UPyA** in the monomer mixture and the final fraction on the polymer backbone, the degree of polymerization of both the **ILA** and the **UPyA**, the calculated theoretical and practical number-averaged molecular weight via ^1^H NMR, and the polydispersity determined by GPC are listed in Table 2.

As shown in Figure 1b, the copolymerization of **ILA** with a 5% feeding molar ratio of **UPyA** at 80 °C with [**ILA** + **UPyA**]/[DBTTC] = 20:1 (green rhombus) showed similar kinetics as the homopolymerization entry 2 (blue circle in Figure 1a), and in 3 h the copolymerization reached its full conversion of 55%. Entry 8 with [**ILA** + **UPyA**]/[DBTTC] = 100:1 also showed comparable kinetics (brown star in Figure 1b) as compared to the homopolymerization (entry 3) under the same conditions (orange triangle in Figure 1a)). As shown in Table 2, when the initial fraction of **UPyA** increased from 5% to 15% and to 25% the overall conversion of the two monomers also increased from 55% to 77% and to 84% respectively (see entry 5–7), this indicated a higher reactivity catalyzed by the HBs in monomer **UPyA**, presumably due to the attractive association of **UPyA** in compared to the repulsive ionic nature of **ILA**. Despite the difficulties of copolymerizing non-ionic and ionic monomers, the control over molecular weight by DBTTC remained moderate in all cases, while control over polydispersity was less pronounced compared to the homopolerizations, which again was anticipated again due to the strong association among UPy moieties and ionic clusters of pyrrolidinium and TFSI ions.

In order to find the proper fraction of UPy on the polymer chain for application as self-healing electrolytes, **CPILU-y** with different UPy contents were prepared. As shown in Figure 1c, as the content of the strong dimerization of UPy moieties increased, the **CPILU-y** altered from a gel (5%) to a glassy solid (25%) and finally to a powdery material (>25%), indicating increased interchain forces due to the quadrupolar HBs of UPy moieties.

### 3.2. Thermal Characterization via Thermogravimetric Analysis (TGA) and Differential Scanning Calorimetry (DSC)

**Figure 2:**
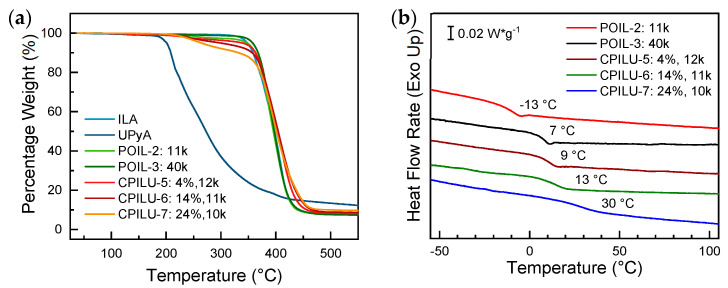
Thermal characterization of ionic monomer **ILA**, HBs monomer **UPyA**, homopolymers **POIL-x**, and copolymers **CPILU-y** via (**a**) thermogravimetric analysis measured at a heating rate of 10 K·min^−1^ under N_2_ atmosphere; and (**b**) differential scanning calorimetry measured at a heating rate of 5 K·min^−1^ with the thermal history cancelled by a preheating/cooling circle (the baseline of **POIL-3** was corrected manually).

To understand the thermal stability of the POILs thermogravimetric analysis (TGA) was employed. The 5%-weight-loss temperature and the degradation onset temperature are listed in Table 3. The ionic monomer **ILA** demonstrated excellent thermal stability up to 348 °C as shown in the Figure 2a, while the non-ionic monomer **UPyA** showed lower stability until 198 °C. For the homopolymers **POIL-2** and **POIL-3** the thermal stabilities were slightly higher than that of the monomer, manifesting a minor influence by molecular weight on the thermal stability. The thermal stabilities of the **POIL-x** are comparable with those of similar polypyrrolidinium-polymers as previously reported [42]. However, when **UPyA** was incorporated into the polymer backbone the thermal stability showed a decreasing trend as the **UPyA** fraction increased. The degradation of the copolymer **CPILU-y** was found to be a two-step process, where the UPy moieties were decomposed first at around 200 °C, followed by the ionic moieties decomposing at around 360 °C (see Table 3, column **Onset *T***/**°C**), indicating a resonable thermal stability compared to the homopolymers.

For the electrolytes used in batteries, it is often observed that the batteries can provide different local temperatures which could introduce a temperature-controlled structural transition of the electrolytes, causing changes in the physical and chemical properties of the electrolytes and further affecting the performance of the batteries, but also reaching temperatures sufficient to activate dynamic self-healing processes. Therefore, the thermal behavior of the polymers in this work was studied by differential scanning calorimetry (DSC). As shown in Figure 2b, the crystallization and melting peaks of the pyrrolidinium moieties [43] were not found in the DSC curves of all POILs, but only glass transitions were observed. This could be advantageous for polymers applied as electrolytes, as a better conductivity value can be achieved both above and below the glass transition temperature in amorphous regions (analogous to higher conductivities achieved in amorphous region in PEO [44]). All *T*_g_s are listed in Table 3 and are also shown in the plot. We found that the molecular weight of the **POIL-x** and the UPy content of the **CPILU-y** indeed influenced the glass transition temperature. When the molecular weight increased from 11 kDa to 40 kDa, *T*_g_ also increased from −13 °C (**POIL-2**) to 7 °C (**POIL-3**), which is potentially indicative of more restricted chain motions. When only 4% of the UPy moieties were introduced into the polymers, *T*_g_ drastically increased from −13 °C to 9 °C, revealing that the dimerizable UPy moieties act as a strong “chain-hardener” in comparison to the increase of molecular weight. If the UPy content increased from 4% to 14% and then to 24%, *T*_g_ increased further from 9 °C to 13 °C and to 30 °C, respectively, this evidenced stiffer chain segments in **CPILU-6** and **CPILU-7** as a result of a denser UPy dimerization.

### 3.3. Conductivity via Broadband Dielectric Spectroscopy (BDS) and Zero Shear Viscosity via Rheology

**Figure 3:**
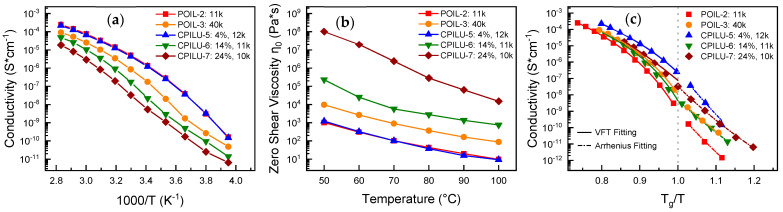
Conductivity *σ* of homo-/copolymer **POIL-x** and **CPILU-y** (**a**) as a function of inverse temperature 1000/T; (**c**) glass transition temperature divided by measurement temperature *T*_g_/*T*; and (**b**) zero shear viscosity *η*_0_ of homo-/copolymer **POIL-x** and **CPILU-y** versus temperature *T*.

To elucidate the potential of the polymers to be applied as electrolytes the DC conductivity was investigated by broadband dielectric spectroscopy (BDS). As shown in Figure 3a the low-molecular-weight **POIL-2** showed a higher conductivity compared to the high-molecular-weight **POIL-3**, matching the conclusions that—in line with expectations—low-molecular-weight POILs generally offer higher conductivity [25,26,27,28]. When 4% UPy moieties were introduced into the polymer backbone (**CPILU-5**), the conductivity was barely influenced by the non-conductive UPy moieties as compared to the homopolymer **POIL-2** (see red squares for **POIL-2** and blue triangles for **CPILU-5** in Figure 3a). However, when the UPy content continued increasing, a generally lower conductivity could be observed within the whole temperature range, as represented by **CPILU-6** and **CPILU-7**. It is often reported [45,46,47] that the conductivity of low-molecular-weight ionic liquids is affected by the viscosity of the conductive system, wherefore the zero shear viscosity *η*_0_ was determined to assist the interpretation of the conductivity behavior of the POILs. As shown in Figure 3b, an inverse relation was observed for the viscosity for those POILs, which can be explained by the higher molecular weight polymers also showing higher viscosity that further hinders ion transport.

In addition to the notable influence of the viscosity, the conductivity of polymer electrolytes before and after the thermal transition is significantly altered. Thus the conductivity of the polymers was plotted versus their glass transition temperature divided by the measurement temperature (*T*_g_/*T*), as shown in Figure 3c. The variation in conductivity of the polymers by temperature showed two distinct regions in the plot. When the temperature was higher than the polymers’ *T*_g_, segmental motion was enabled, and the conductivity was coupled with the segmental motion, thus following the Vogel−Fulcher−Tammann (VFT) equation. At temperatures below *T*_g_, segments were frozen, and the conductivity plot displayed Arrhenius behavior, which is comparable to the conductive behavior of inorganic glasses [48]. As previously reported [49,50], if the conductivity at *T*_g_ is roughly equal to 10^–15^ S·cm^−1^ in an ionic system, the charge transport is then fully governed by the structural relaxation of the system, which is segmental motion in case of polymer electrolytes. For our polymers, the conductivities at *T*_g_, as listed in Table 4, are all higher than the reported threshold value (10^–15^ S·cm^−1^) for the fully relaxation-coupled systems. As a consequence, the polymers in this work exhibited a certain degree of decoupled ion transport from the segmental dynamics. To clearly visualize this behavior, the decoupling index defined as *R*_σ, Tg_ = 15 + log *σ*_Tg_ [51,52] was applied. Among our copolymers, those polymer with the strongest decoupling, ie those where the charge transport is least linked to the segmental relaxation, is the copolymer **CPILU-5** with 4% UPy moieties, with a decoupling index of 8.4. The least-decoupled homopolymer can be observed in the case of the homopolymer **POIL-2** (for *R*_σ, Tg_ of other polymers, see Table 4). Because the copolymers show a higher decoupling index, they could provide better conductivity and mechanical strength simultaneously. Therefore, a tensile test was employed to explore their mechanical strength and their self-healing ability, which is discussed in next chapter.

### 3.4. Mechanical and Self-Healing Characterization via Tensile Test

**Figure 4:**
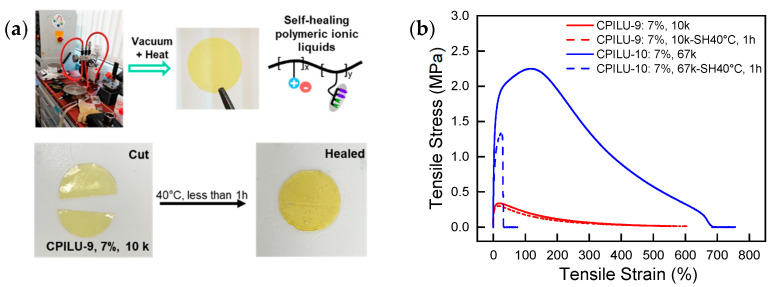
(**a**) Visualized self-healing of **CPILU-9**; and (**b**) stress–strain curves of pristine (solid line) and self-healed (at 40 °C for 1 h, dash line) **CPILU-9** and **CPILU-10** at a strain rate of 20 mm·min^−1^ at room temperature.

To evaluate the mechanical strength and the prominent self-healing ability, two copolymers, both with 7% UPy content but with different molecular weights, were synthesized, which are designated as **CPILU-9** (7% UPy, 10 kDa) and **CPILU-10** (7% UPy, 67 kDa) (for synthesis details, see Supplementary Material Section S1.2). The copolymers were hot-vacuum-pressed into bars or disc specimens, and self-healing ability tests were firstly performed with **CPILU-9**, as visualized in Figure 4a. A specimen was cut vertically at the long edge into two pieces and brought back with the freshly generated surface tightly and accurately matched. Owing to the quadrupolar HBs of the UPy moieties associating at the edge of the wound, self-healing was observed within 1 h at 40 °C or 24 h at room temperature. To quantify the mechanical strength and the self-healing ability, tensile tests of both pristine and self-healed bar specimens of **CPILU-9** and **CPILU-10** were performed at a strain rate of 20 mm·min^−1^ at room temperature, as shown in Figure 4b.

For the pristine specimen, increasing the molecular weight from 10 kDa to 67 kDa indeed drastically enhanced the mechanical strength from 0.34 MPa (**CPILU-9**, red solid line in Figure 4b) to 2.25 MPa (**CPILU-10**, blue solid line in Figure 4b) in terms of ultimate stress. Both specimen showed good ductility, with roughly 550% and 650% in relation to the tensile strain for **CPILU-9** and **CPILU-10**, respectively. Regarding self-healing ability, the self-healed specimen of **CPILU-9** showed an almost identical tensile behavior (red dash line in Figure 4b) in comparison to the pristine specimen, with a slightly reduced ultimate stress (0.30 MPa) and a similar ductility. These results indicated efficient self-healing (88% in terms of ultimate stress compared to the pristine specimen) at 40 °C for 1 h which is the consequence of the flexible segments and the strong quadrupolar HBs of UPy moieties in **CPILU-9**. As for **CPILU-10**, with a molecular weight of 67 kDa, the self-healed specimen generated an ultimate stress of 1.33 MPa (59% in terms of ultimate stress compared to the pristine specimen) and a poor ductility at 32% tensile strain. This could be attributed to the more entangled segments in the high-molecular-weight **CPILU-10** which hindered self-healing by UPy dimerization.

## 4. Conclusions

Novel polymeric ionic liquids **POIL-x** and copolymers **CPILU-y** bearing quadrupolar hydrogen bonds were synthesized via RAFT polymerization using DBTTC as chain transfer agent. Kinetic studies revealed that the HBs monomer **UPyA** showed preferable incorporation in a copolymerization of **ILA**, anticipated as the attractive association of **UPyA**. While the synthesized homopolymers were thermally stable up to 360 °C, the introduction of UPy moieties into the polymer chains decreased the thermal stability and altered the decomposition into a two-step process. Examining a combination of data from DSC, BDS, and rheology, a decoupling of conductivity from the glass transition was observed in all polymers, while the trend of the zero shear viscosity values of all the polymers matched that of their conductivity values. A copolymer with 4% UPy content displayed the highest decoupling index of 8.4, proving that both, self-healing and excellent conductivities can be embedded into the same type of a POIL using complex hydrogen-bonding. While copolymers with low molecular weights demonstrated both better conductivity and self-healing efficiency, the high-molecular-weight samples showed higher ultimate strength but lower healing efficiency and conductivity. This work proves that HBs are a useful tool for designing electrolytes with unique properties like decoupled conductivity and self-healing ability, which can contribute to battery science and electronic devices such as supercapacitors in the future.

## Figures and Tables

**Table 1:** Data from RAFT polymerizations of **ILA** and the resulting homopolymer **POIL-x**.

Sample	Entry	M/CTA ^a^	*T*/°C	*t*/h	*conv.* ^b^	*M*_n, th_^c^	*M*_n, NMR_	PDI ^d^
**POIL-**	1	20:1	70	7	38%	3900	8000	1.26
	2	20:1	80	7	62%	6100	11,000	1.26
	3	100:1	80	7	67%	32,500	40,100	1.34
	4	200:1	80	7	79%	75,800	82,100	1.36

^a^ Polymerization was carried out in DMF with AIBN as the initiator, and the ratio of [DBTTC]/[AIBN] was kept to 1:0.1 while the monomer concentration was kept at 1 mmol monomer in 1 mL DMF; ^b^ conversion was detected by ^1^H NMR using trioxane as reference; ^c^ the number-average molecular weight calculated by the equation: *M*_n,th_ = *conv.* × ([M_ILA_]/[DBTTC]) × *m*_ILA_ + *m*_DBTTC_; ^d^ PDI was determined by DMF + LiTFSI (0.1 M) GPC with a PS standard.

**Table 2:** Data from RAFT copolymerizations of **ILA** with **UPyA** and the resulting copolymer **CPILU-y**.

Sample	Entry	M/CTA ^a^	*f*_UPy_ ^b^	*T*/°C	*t*/h	*conv.* ^c^	*F*_UPy, NMR_ ^d^	DP_IL_ ^e^	DP_UPy_ ^f^	*M*_n, th_ ^g^	*M*_n, NMR_	PDI ^h^
**CPILU-**	5	20:1	5%	80	7	55%	4%	22.5	0.9	5400	11,800	1.35
	6	20:1	15%	80	7	77%	14%	18.6	2.6	7200	10,700	1.32
	7	20:1	25%	80	7	84%	24%	18.7	4.5	7400	10,500	1.25
	8	100:1	5%	80	7	65%	4%	90.1	3.6	30,700	44,400	1.36

^a^ Polymerization was carried out in DMF with AIBN as the initiator, and the ratio of [DBTTC]/[AIBN] was kept to 1:0.1, while the monomer concentration was kept at 1 mmol monomer in 1 mL DMF; ^b^ *f*_UPy_ was the initial molar fraction of **UPyA** in the monomer mixture; ^c^ conversion was detected by ^1^H NMR using trioxane as reference; ^d^ *F*_UPy_ was the final molar fraction of **UPyA** on the polymer backbone, calculated from ^1^H NMR; ^e, f^ calculated from relative integral of protons from ^1^H NMR (for details see Supplementary Material Section S1.2); ^g^ the number-average molecular weight calculated by the equation: *M*_n,th_ = *conv.* × [([M_ILA_]/[DBTTC]) × *m*_ILA_ + ([M_UPyA_]/[DBTTC]) × *m*_UPyA_] + *m*_DBTTC_; ^h^ PDI was determined by DMF + LiTFSI (0.1 M) GPC with PS standard.

**Table 3:** Thermal characterization data of **ILA**, **UPyA**, **POIL-x**, **and CPILU-y** via thermogravimetric analysis and differential scanning calorimetry.

Sample	Entry	Sample Info.	5 wt%-loss *T*/°C	Onset *T*/°C	*T*_g, DSC_/°C ^a^
**ILA**	-	monomer	348	348	-
**UPyA**	-	monomer	201	198	-
**POIL-**	2	11 k	348	353	−13
	3	40 k	360	357	7
**CPILU-**	5	4%, 12 k	338	198, 360	9
	6	14%, 11 k	297	212, 365	13
	7	24%, 10 k	263	215, 363	30

^a^ Glass transition temperature via DSC.

**Table 4:** Glass transition temperature and conductivity at glass transition temperature, and zero shear viscosity and conductivity at 80 °C of **POIL-x** and **CPILU-y**.

Sample	Entry	Sample Info.	*T*_g, DSC_/°C ^a^	*σ*_Tg_/S·cm^−1^^b^	*R_σ_*_, *T*g_ ^c^	*η*_0, 80 °C_ Pa·s ^d^	*σ*_80 °C_/S·cm^−1^^e^
**POIL-**	2	11 k	−13	3.07 × 10^−9^ (−10 °C)	6.5	43	2.46 × 10^−4^
	3	40 k	7	2.04 × 10^−8^ (10 °C)	7.3	368	9.09 × 10^−5^
**CPILU-**	5	4%, 12 k	9	2.55 × 10^−7^ (10 °C)	8.4	37	2.19 × 10^−4^
	6	14%, 11 k	13	2.19 × 10^−8^ (20 °C)	7.3	2.76 × 10^3^	4.85 × 10^−5^
	7	24%, 10 k	30	3.27 × 10^−8^ (30 °C)	7.5	2.84 × 10^5^	1.84 × 10^−5^

^a^ Glass transition temperature via DSC; ^b^ conductivity at glass transition temperature; ^c^ decoupling index calculated with *R*_σ, Tg_ = 15 + log *σ*_Tg_; ^d^ zero shear viscosity at 80 °C; ^e^ and conductivity at 80 °C.

## Data Availability

Not applicable.

## Supplementary Materials

The following supporting information can be downloaded at: https://www.mdpi.com/article/10.3390/polym14194090/s1, Scheme S1: Synthesis route of monomers, RAFT chain transfer agent, and polymers; Scheme S2: Synthesis rout of POILC-z for GPC calibration; Figure S1–S4: GPC curves of all the polymers; Figure S5–S12 NMR spectra of all the compounds and polymers; Table S1: Synthesis data of the copolymers CPILU-9 and CPILU-10 for tensile test; Table S2: GPC data of precursor polymers pCPA and final POILC-z, planned for calibration; Table S3: Conductivity of POIL-x and CPILU-y from −20 °C to 80 °C, measured by BDS; Table S4: Zero shear viscosity of POIL-x and CPILU-y from 50 °C to 100 °C.
