# Supplementary material for: Synthesis and Characterization of Quadrupolar-Hydrogen-Bonded Polymeric Ionic Liquids for Potential Self-Healing Electrolytes
Source: Polymers (Basel). 2022 Sep 29;14(19):4090. doi: 10.3390/polym14194090 (PMC9572781; doi:10.3390/polym14194090)
Supplement: Supplementary file 1 [file polymers-14-04090-s001.zip › polymers-1914198-supplementary.pdf]

Supporting Information

for

Synthesis and Characterization of Quadrupolar-Hydrogen-Bonded Polymeric Ionic Liquids for Potential Self-Healing Electrolytes

Chenming Li [1], Rajesh Bhandary [1], Anja Marinow [1], Dmitrii Ivanov [1], Mengxue Du [2], René Androsch [2], Wolfgang H. Binder [1,*]

[1] Macromolecular Chemistry, Institute of Chemistry, Faculty of Natural Science II, Martin Luther University Halle-Wittenberg, Von-Danckelmann-Platz 4, 06120 Halle (Saale), Germany

[2] Interdisciplinary Center for Transfer-oriented Research in Natural Sciences, Martin Luther University Halle-Wittenberg, 06099 Halle (Saale), Germany

* Correspondence: wolfgang.binder@chemie.uni-halle.de (W.H.B.)

Table of Content

1. EXPERIMENTAL PROCEDURE ···· **1**

1.1. Materials and Methods ···· **1**

1.2. Synthesis of Monomers and Polymers ···· **2**

a) Synthesis of 3-Chloropropyl Acrylate (**CPA**) ···· 2

b) Synthesis of 1-(3-(Acryloyloxy)propyl)-1-methylpyrrolidin-1-ium Bis(trifluoromethyl sulfonyl)imide (**ILA**) ···· 2

c) Synthesis of 2-(3-(6-Methyl-4-oxo-1,4-dihydropyrimidin-2-yl)ureido)ethyl Acrylate (**UPyA**) 3

d) Synthesis of Dibenzyl Carbonotrithioate (**DBTTC**) ···· 4

e) RAFT Homopolymerization of **ILA** for **POIL-x** ···· 4

f) RAFT Copolymerization of **ILA** with **UPyA** for **CPILU-y** ···· 4

1.3. Synthesis of POILC-z for GPC Calibration ···· **5**

g) RAFT Polymerization of **CPA** for **pCPA** ···· 5

h) Quaternization and Ion Exchange of **pCPA** for **POILC-z** ···· 5

2. GPC CURVES AND DATA ···· **7**

3. CONDUCTIVITY AND VISCOSITY DATA ···· **9**

4. NMR SPECTRA ···· **10**

5. REFERENCE ···· **16**

1. Experimental Procedure

1.1. Materials and Methods

Solvents

Acetonitrile (MeCN), dimethyl sulfoxide (DMSO) and dimethyl formamide (DMF) were purchased from Grüssing (Filsum, Germany); methylene chloride (DCM), acetone and *n*-hexane were purchased from Overlack (OQEMA GmbH, Mönchengladbach, Germany); tetrahydrofuran (THF) was purchased from Carl Roth (Karlsruhe, Germany); and dimethyl sulfoxide 99.7+% extra dry was purchased from Fisher Scientific (Schwerte, Germany). All the volatile solvents were purchased in technical grade and redistilled at least once before use. The DMF for polymerization was dried over a solvent purification system and the dimethyl sulfoxide 99.7+% extra dry was used as received. NMR solvents deuterated chloroform (chloroform-*d*) and deuterated dimethyl sulfoxide (DMSO-d_6) were purchased from Chemotrade (Eckert & Ziegler Gruppe, Düsseldorf, Germany) and used as received.

Methods

Nuclear Magnetic Resonance

All ^{1}H-NMR and ^{13}C-NMR spectra were measured on a Varian FT-NMR spectrometer (500 and 101 MHz, respectively) (Agilent Technologies Germany GmbH & Co. KG, Waldbronn, Germany). All samples were measured at 27 °C using deuterated chloroform (chloroform-*d*) or deuterated dimethyl sulfoxide (DMSO-d_6). Chemical shifts (δ) were recorded in parts per million (ppm) relative to the remaining solvent signals (chloroform-*d*: 7.26 ppm (^{1}H) and 77.0 ppm (^{13}C), DMSO-d_6: 2.50 ppm (^{1}H) and 39.5 ppm (^{13}C). Chemical shifts were reported with the following notations: s, singlet; d, doublet; t, triplet; q, quartlet; p, pentlet; m, a more complex multiplet or overlapping multiplets. The data analysis was performed via MestReNova (version 9.0.1-13254).

Gel Permeation Chromatography

GPC (THF) measurements were performed on a Viscotek GPCmax VE 2002 (Malvern Panalytical GmbH, Kassel, Germany) GPC machine with a CLM3008 (Tguard, Org Guard 10×4.6) and a CLM3011 (Range:1000 – 400000 g·mol^{-1}) main column at 30 °C. Detection of the refractive index was realized via a VE 3580 RI detector (Malvern Panalytical GmbH, Kassel, Germany) at 35 °C. Poly(styrene) (PS) was used as the external calibration standards with a molecular weight range from 300 to 170000 g·mol^{-1}. GPC (DMF+0.1M LiTFSI) measurements were performed on a Viscotek GPCmax VE 2001 (Malvern Panalytical GmbH, Kassel, Germany) GPC machine with a CLM3008 precolumn and a GMH$_{HR}$-N-18055 main column in DMF with LiTFSI (0.1 M) at 60 °C. Detection of the refractive index was realized via a VE 3580 RI detector (Malvern Panalytical GmbH, Kassel, Germany) at 35 °C. Poly(styrene) (PS) was used as the external calibration standards with a molecular weight range from 1050 to 115000 g·mol^{-1}. The data analysis was performed via OminSEC4.5 and Origin 2018 (version b9.5.0.193).

1.2. Synthesis of Monomers and Polymers

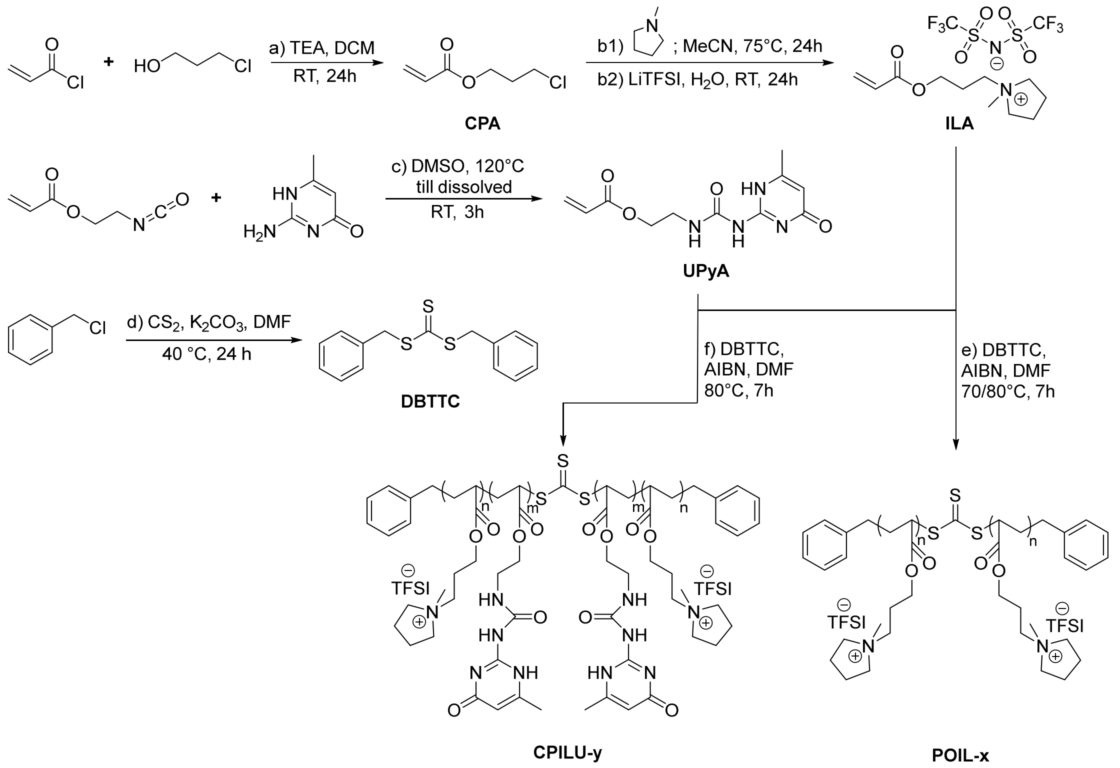

Scheme S1 Synthetic route of monomers, RAFT chain transfer agent, and polymers.

a) *Synthesis of 3-Chloropropyl Acrylate (**CPA**)*

The synthesis was adopted from the reference [1] with slight modification. 3-Chloro-1-propanol (20.0 mL, 236.3 mmol) was dissolved in 150 mL dry DCM in a three neck flask and cooled at 0 °C. Triethylamine (33.3 mL, 236.3 mmol) dissolved in 75 mL dry DCM was added dropwise via a separation funnel under vigorous stirring over 1 hour. Then acryloyl chloride (18.3 mL, 214.8 mmol) dissolved in 75 mL dry DCM was added in the same way as triethylamine. The reaction mixture was stirred at 0 °C for about 1 hour and subsequently at room temperature for 24 hours. After the reaction was finished, the reaction mixture was filtered and the organic liquid was washed with 1 M hydrochloric acid (150 mL × 1), DI water (150 mL × 3), and brine (150 mL × 1). The organic layer was collected, dried over sodium sulfate, filtered, and concentrated under reduced pressure. The crude product was then distilled at 55 °C (vapor temperature = 31 °C) at 0.015 mbar. The purified 3-chloropropyl acrylate (**CPA**) was afforded as colorless liquids with the yield of 27.7 g (87 %). ^{1}H NMR (400 MHz, Chloroform-*d*) δ 6.41 (dd, J = 17.4, 1.5Hz, 1H), 6.12 (dd, J = 17.4, 10.4 Hz, 1H), 5.84 (ddd, J = 10.5, 1.5, 0.5 Hz, 1H), 4.31 (t, J = 6.1 Hz, 2H), 3.63 (t, J = 6.4 Hz, 2H), 2.14 (p, J = 6.3 Hz, 2H).^{13}C NMR (101 MHz, Chloroform-*d*) δ 165.95, 130.94, 128.17, 61.20, 41.13, 31.59.

b) *Synthesis of 1-(3-(Acryloyloxy)propyl)-1-methylpyrrolidin-1-ium Bis(trifluoromethanesulfonyl)imide (**ILA**)*

The synthesis was adopted from the reference [1] with slight modification and was carried out in two steps:

First step b1):

CPA (7.5 g, 50.7 mmol) was dissolved in MeCN (30 mL) and heated to 75 °C, followed by adding 1-methylpyrrolidine (5.6 mL, 53.3 mmol). After 24 hours, the reaction mixture was cooled down to room temperature and MeCN was removed by a rotary evaporator at 35 °C. Then a little amount of water (5 to 10 mL) was added to dissolve the raw product and the water solution was washed with DCM (50 mL × 3), whereafter the water phase was concentrated under reduced pressure. The crude product was afforded as a viscous brownish oil and was used directly in next step without further purification.

Second step b2):

The product from the *first step b1)* was dissolved in water (10 mL) and added to a water solution (10 mL) of lithium bis(trifluoromethanesulfonyl)imide (15.5 g, 53.3 mmol). The reaction mixture was then stirred at room temperature for 24 hours. After the reaction was finished, the reaction mixture was extracted with DCM (50 mL × 3). Then the combined organic layers were washed with water (50 mL × 3) and dried over sodium sulfate. The sodium sulfate was filtered and active charcoal was added to the organic solution and stirred at room temperature for 5 hours to decolorize the product. After decolorization the active charcoal was filtered and DCM was evaporated under reduced pressure. The product 1-(3-(acryloyloxy)propyl)-1-methylpyrrolidin-1-ium bis(trifluoromethanesulfonyl)imide (**ILA**) was afforded as a transparent viscous yellowish oil with the total yield of 31.9 g (80%). ^{1}H NMR (400 MHz, DMSO-d_6) δ 6.38 (dd, J = 17.3, 1.5 Hz, 1H), 6.18 (dd, J = 17.3, 10.4 Hz, 1H), 5.99 (dd, J = 10.4, 1.5 Hz, 1H), 4.19 (t, J = 6.2 Hz, 2H), 3.55 – 3.37 (m, 6H), 3.00 (s, 3H), 2.20 – 2.01 (m, 6H). ^{13}C NMR (101 MHz, DMSO-d_6) δ 165.74, 132.34, 128.48, 124.73, 121.53, 118.32, 115.12, 64.03, 61.74, 60.78, 47.97, 23.30, 21.51. ^{19}F NMR (376 MHz, DMSO-d_6) δ -78.74.

c) *Synthesis of 2-(3-(6-Methyl-4-oxo-1,4-dihydropyrimidin-2-yl)ureido)ethyl Acrylate (**UPyA**)*

The synthesis was adopted from the reference [2] with slight modification. 6-Methylisocytosine (5.0 g, 40.0 mmol) was placed in 100 mL DMSO and the mixture was heated at 120 °C till a clear solution was obtained. Then the flask was removed from the oil bath and 2-isocyanatoethyl acrylate (5.2 mL, 42.0 mmol) was added immediately to the flask, followed by cooling in a water bath to inhibit polymerization. The reaction mixture was stirred for 3 hours at room temperature. After reaction was finished, the white solid was filtered and washed thoroughly with *n*-hexane and dried under reduced pressure. Then the product was dried via a freeze-drier to remove residual DMSO. The purified product was afforded as a white powder with a yield of 9.5 g (87 %). ^{1}H NMR (400 MHz, Chloroform-*d*) δ 12.95 (s, 1H), 11.94 (s, 1H), 10.47 (s, 1H), 6.44 (dd, J = 17.4, 1.5 Hz, 1H), 6.13 (dd, J = 17.3, 10.4 Hz, 1H), 5.88 – 5.70 (m, 2H), 4.29 (t, J = 5.7 Hz, 2H), 3.57 (q, J = 5.6 Hz, 2H), 2.23 (s, 2H). ^{13}C NMR (126 MHz, Chloroform-*d*) δ 173.00, 166.22, 156.97, 154.68, 148.39, 131.00, 128.52, 106.93, 63.03, 38.96, 19.11.

*d) Synthesis of Dibenzyl Carbonotrithioate (**DBTTC**)*

The synthesis was performed according to reference [3]. ^{1}H NMR (400 MHz, Chloroform-*d*) δ 7.40 – 7.24 (m, 10H), 4.64 (s, 4H). ^{13}C NMR (101 MHz, Chloroform-*d*) δ 222.86, 135.07, 129.39, 128.84, 127.91, 41.68.

*e) RAFT Homopolymerization of **ILA** for **POIL-x***

RAFT homopolymerization of **ILA** was carried out in DMF using **DBTTC** as the RAFT chain transfer agent and ABIN as the initiator. Typically, **ILA** (1086.0 mg, 2.3 mmol), **DBTTC** (5.6 mg, 2.3×10^{-2} mmol), AIBN (0.4 mg, 2.3×10^{-3} mmol) were dissolved in DMF (2.3 mL) inside a Schlenk tube. Trioxane was added as the NMR reference to monitor the monomer conversion. The Schlenk tube was sealed with a rubber septum and the reaction mixture was purged with nitrogen for 30 minutes to deoxygenate under vigorous stirring. The tube was then quickly immersed in an oil bath thermostated at 80 °C. Aliquots were taken at certain time intervals and measured ^{1}H NMR to study the kinetics of the polymerization by calculating the integral of vinylenic protons of **ILA** to the integral of trioxane protons. The reaction was stopped by opening the septum and subsequently precipitated into DCM twice to eliminate the monomer residue. The polymer was collected and finally dried at 80 °C under vacuum for 24 hours. The molecular weight was evaluated by ^{1}H NMR using the characteristic methyl protons of the pendent pyrrolidinium (≡N-CH_3, 3nH, δ = 3.02 ppm, with n being the degree of polymerization) and the characteristic aromatic protons of **DBTTC** (ph*H*, 10H, δ = 7.37 ppm).

*f) RAFT Copolymerization of **ILA** with **UPyA** for **CPILU-y***

RAFT copolymerization of **ILA** with **UPyA** was carried out in DMF using **DBTTC** as the RAFT chain transfer agent and ABIN as the initiator. Typically, **ILA** (1043.0 mg, 2.2 mmol), and **UPyA** (29 mg, 0.109 mmol) were placed in DMF (2 mL) in a Schlenk tube. The tube was sealed with a rubber septum and the reaction mixture was purged with nitrogen for 30 minutes to deoxygenate under vigorous stirring. After deoxygenation the tube was heated in an oil bath at 120 °C till a clear solution was obtained, then the temperature of the oil bath was cooled to 80 °C. In the meantime, to a flask **DBTTC** (33.2 mg, 1.1×10^{-1} mmol), AIBN (1.88 mg, 1.1×10^{-2} mmol), and trioxane as the NMR reference were dissolved in deoxygenated DMF (0. 29 mL), and this solution was transferred to the reaction Schlenk tube by a syringe to initiate the polymerization. Aliquots were taken at certain time intervals and measured ^{1}H NMR to study the kinetics of the polymerization by calculating the integral of vinylenic protons of **ILA** and **UPyA** to the integral of trioxane protons. The reaction was stopped by opening the septum and subsequently precipitate into DCM thrice to remove the monomer residue. The polymer was collected and finally dried at 80 °C under vacuum for 24 hours. The molecular weight was evaluated by ^{1}H NMR using the characteristic methyl protons of the pendent pyrrolidinium (≡N-CH_3, 3nH, δ = 3.02 ppm, with n being the degree of polymerization of **ILA**), the characteristic vinylenic proton of the pendent UPy moiety (-(CH3-)C=C*H*-, mH, δ = 5.80 ppm, with m being the degree of polymerization of **UPyA**), and the characteristic aromatic protons of **DBTTC** (ph*H*,

10H, δ = 7.36 ppm).

Table S1 Synthetic data of the copolymers **CPILU-9** and **CPILU-10** for tensile test.

Sample	Entry	M/CTA	Solvent	$M_{mon.}$/M	f_{UPy}	T	t	conv.	$F_{UPy,NMR}$	DP_{IL}	DP_{UPy}	$M_{n,th}$	$M_{n,NMR}$	$M_{n,GPC}$	PDI
cpILU	**9**	20:1	DMSO	1	8%	80°C	16	97%	7%	20.7	1.4	9540	10452	2985	1.32
	**10**	200:1	DMSO	1	8%	80°C	16	98%	7%	133.8	9.4	96380	66798	3454	1.52

1.3. Synthesis of POILC-z for GPC Calibration

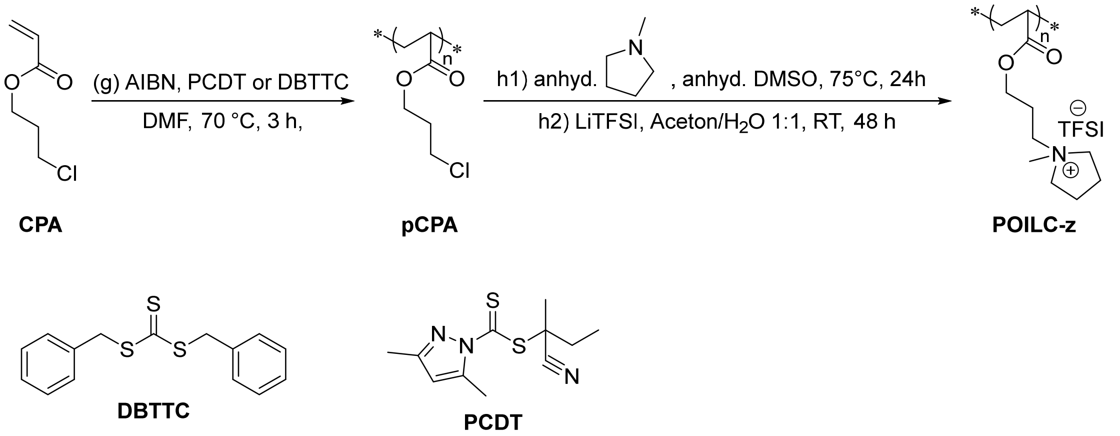

Scheme S2 Synthesis rout of POILC-z for GPC calibration.

*g) RAFT Polymerization of **CPA** for **pCPA***

RAFT polymerization of **CPA** was carried out in DMF using **DBTTC** as the RAFT chain transfer agent and ABIN as the initiator. Typically, **CPA** (447.0 mg, 3.0 mmol), **PCDT** (8.03 mg, 3.0×10^{-2} mmol) AIBN (0.5 mg, 3.0×10^{-3} mmol) were dissolved in DMF (4.5 mL) in a Schlenk tube. The Schlenk tube was sealed with a rubber septum and the reaction mixture was purged with nitrogen for 30 minutes to deoxygenate under vigorous stirring. The tube was then heated in an oil bath thermostated at 70 °C. The reaction was stopped by opening the septum and subsequently precipitated into MeOH twice to eliminate the monomer residue. The molecular weight was evaluated by THF GPC with PS standard.

*h) Quaternization and Ion Exchange of **pCPA** for **POILC-z***

The synthesis was adopted from references [4-6] with modification and was carried out in two steps:
First step h1):
In a glovebox, **pCPA**(122.0 mg, 0.8 mmol, calculated based on the molecular weight of repeating unit) and pre-distilled anhydrous 1-methylpyrrolidine (0.5 mL, 4.1 mmol) were dissolved in anhydrous DMSO (1.0 mL) in a vial. The vial was sealed and heated at 75 °C for 24 hours. After reaction was finished, the reaction mixture was precipitated into THF twice. The crude product was afforded as viscous oil due to its hygroscopic nature and was used directly in next step without further purification.

Second step h2):

The product from *first step h1)* was dissolved in water (1.0 mL) and added to the water solution (1.0 mL) of lithium bis((trifluoromethyl)sulfonyl)imide (2.3 g, 8.0 mmol). After precipitation was observed, acetone (2.0 mL) was added to reach a homogeneous reaction mixture. The reaction mixture was then stirred at room temperature for 24 hours. After reaction was finished, the reaction mixture was precipitated into DCM thrice. The polymer was collected and finally dried at 80 °C under vacuum for 24 hours.

2. GPC Curves and Data

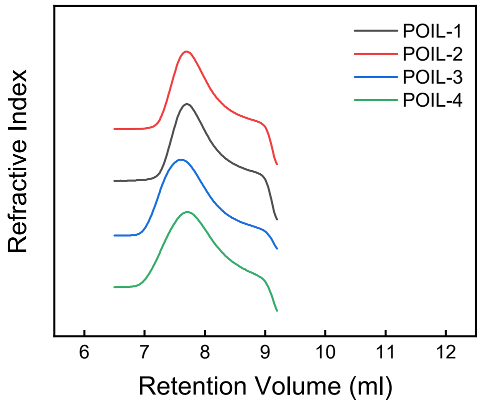

Figure S1 GPC (DMF+LiTFSI, 0.1 M) curves of **POIL-x** using a PS standard calibration.

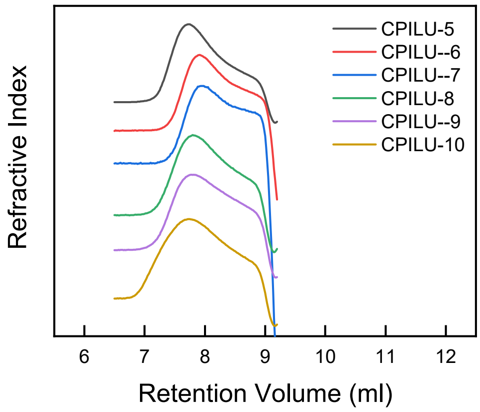

Figure S2 GPC (DMF+LiTFSI, 0.1 M) curves of **CPILU-y** using a PS standard calibration.

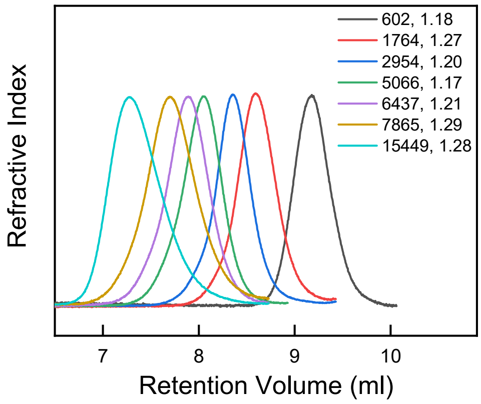

Figure S3 GPC (THF) curves of precursor polymer pCPA using a PS standard calibration.

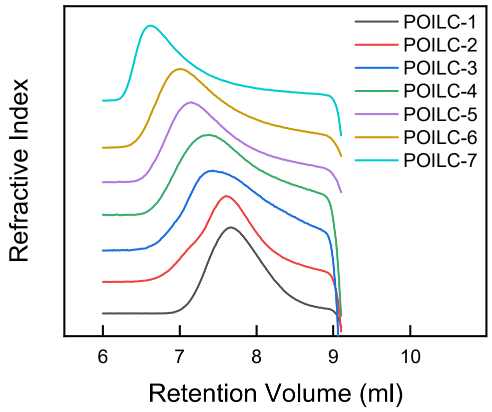

Figure S4 GPC (DMF+LiTFSI, 0.1 M) curves of **POILC-z** supposed to be calibration.

Table S2 GPC data of precursor polymers **pCPA** and final **POILC-z** supposed to be calibration.

**pCPA (in THF)**	**Peak Rv /mL**	**Mn (PS-calibration)**	**PDI**	**Mp**	**DP, Mp**
**pCPA-1**	9.17	600	1.18	690	2.3
**pCPA-2**	8.59	1800	1.27	2100	10.2
**pCPA-3**	8.35	3000	1.2	3300	18.2
**pCPA-4**	8.05	5100	1.17	5300	32.4
**pCPA-5**	7.89	6400	1.21	6900	41.6
**pCPA-6**	7.70	7900	1.29	9500	51.2
**pCPA-7**	7.28	15400	1.28	21100	102.0
**Calibration POILC-z (in DMF+0.1 M LiTFSI)**	**Peak Rv /mL**	**Mn (PS-calibration)**	**PDI**	**Mp (calculated based on DP, Mp of pCPA)**	
**POILC-1**	7.67	4200	1.26	1400	
**POILC-2**	7.60	4500	1.49	5100	
**POILC-3**	7.40	4300	1.73	9000	
**POILC-4**	7.37	4700	1.68	15700	
**POILC-5**	7.13	5500	1.81	20200	
**POILC-6**	7.00	6200	1.99	24800	
**POILC-7**	6.62	9200	2.12	49100	

3. Conductivity and Viscosity Data

Table S3 Conductivity of **POIL-x** and **CPILU-y** from -20 °C to 80 °C, measured by BDS

		Conductivity σ / S*cm^{-1}										
**Sample**	**Entry**	**-20 °C**	**-10 °C**	**0 °C**	**10 °C**	**20 °C**	**30 °C**	**40 °C**	**50 °C**	**60 °C**	**70 °C**	**80 °C**
POIL-x	2	1.64E-10	3.07E-09	3.89E-08	2.80E-07	1.35E-06	5.03E-06	1.43E-05	3.45E-05	7.56E-05	1.46E-04	2.46E-04
	3	4.86E-11	2.67E-10	1.71E-09	2.04E-08	1.75E-07	8.56E-07	3.49E-06	1.03E-05	2.52E-05	5.32E-05	9.09E-05
CPILU-y	5	1.49E-10	3.24E-09	3.68E-08	2.55E-07	1.22E-06	4.40E-06	1.27E-05	3.02E-05	6.61E-05	1.26E-04	2.19E-04
	6	1.38E-11	9.48E-11	4.98E-10	2.90E-09	2.19E-08	1.72E-07	9.03E-07	3.52E-06	1.05E-05	2.57E-05	4.85E-05
	7	6.42E-12	2.50E-11	1.72E-10	1.10E-09	5.43E-09	3.27E-08	1.95E-07	8.42E-07	2.89E-06	8.05E-06	1.84E-05

Table S4 Zero shear viscosity of **POIL-x** and **CPILU-y** from 50 °C to 100 °C.

		Zero Shear Viscosity η_0 Pa*s					
**Sample**	**Entry**	**50 °C**	**60 °C**	**70 °C**	**80 °C**	**90 °C**	**100 °C**
POIL-x	2	1.01E3	301	102	43	20	10
	3	9.57E3	2.63E3	896	368	163	87
CPILU-y	5	1.20E3	323	102	37	16	9
	6	2.30E5	2.52E4	5.72E3	2.76E3	1.35E3	755
	7	1.03E8	1.99E7	2.42E6	2.84E5	6.30E4	1.51E4

4. NMR Spectra

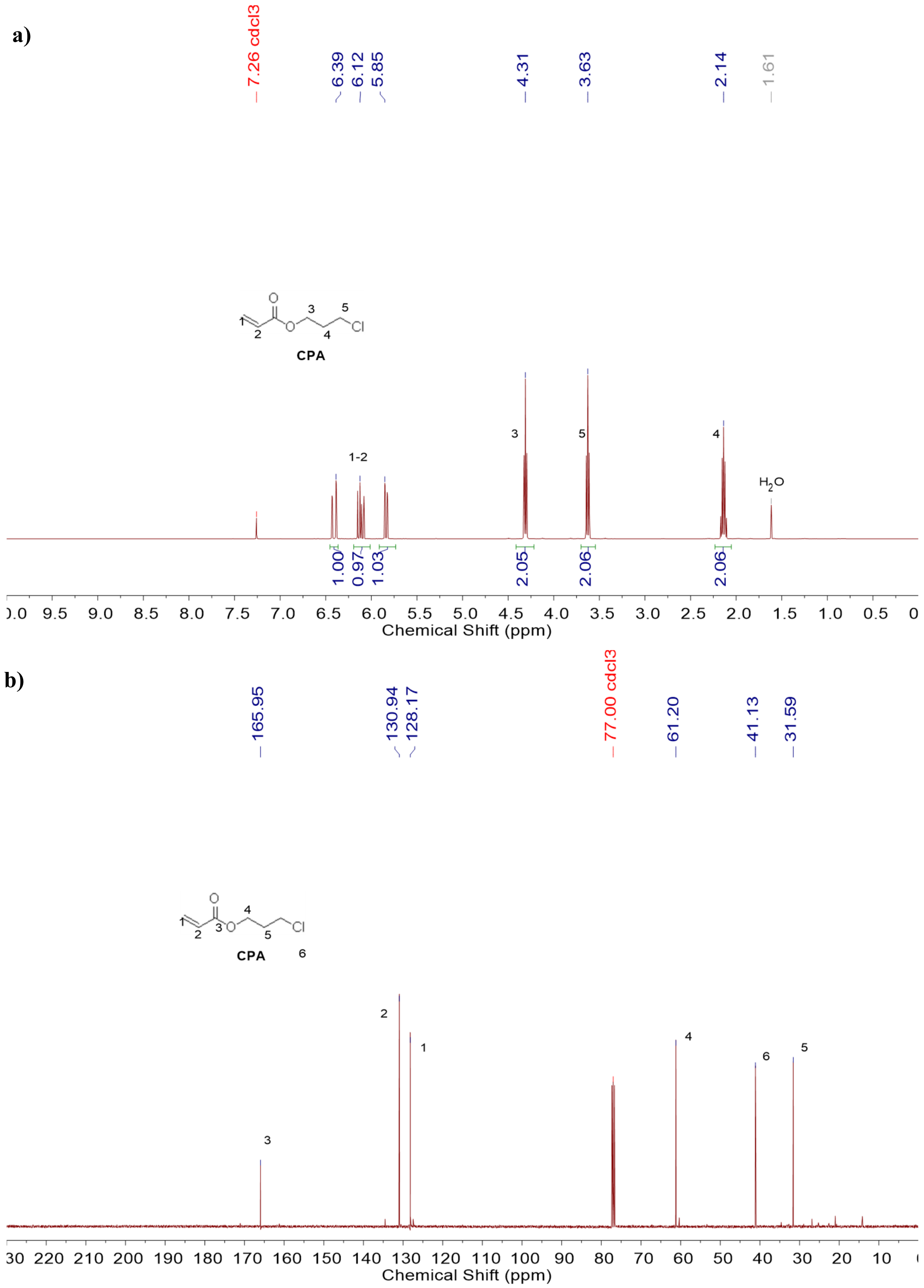

Figure S5 a) ^{1}H and b) ^{13}C NMR spectra of compound **CPA**.

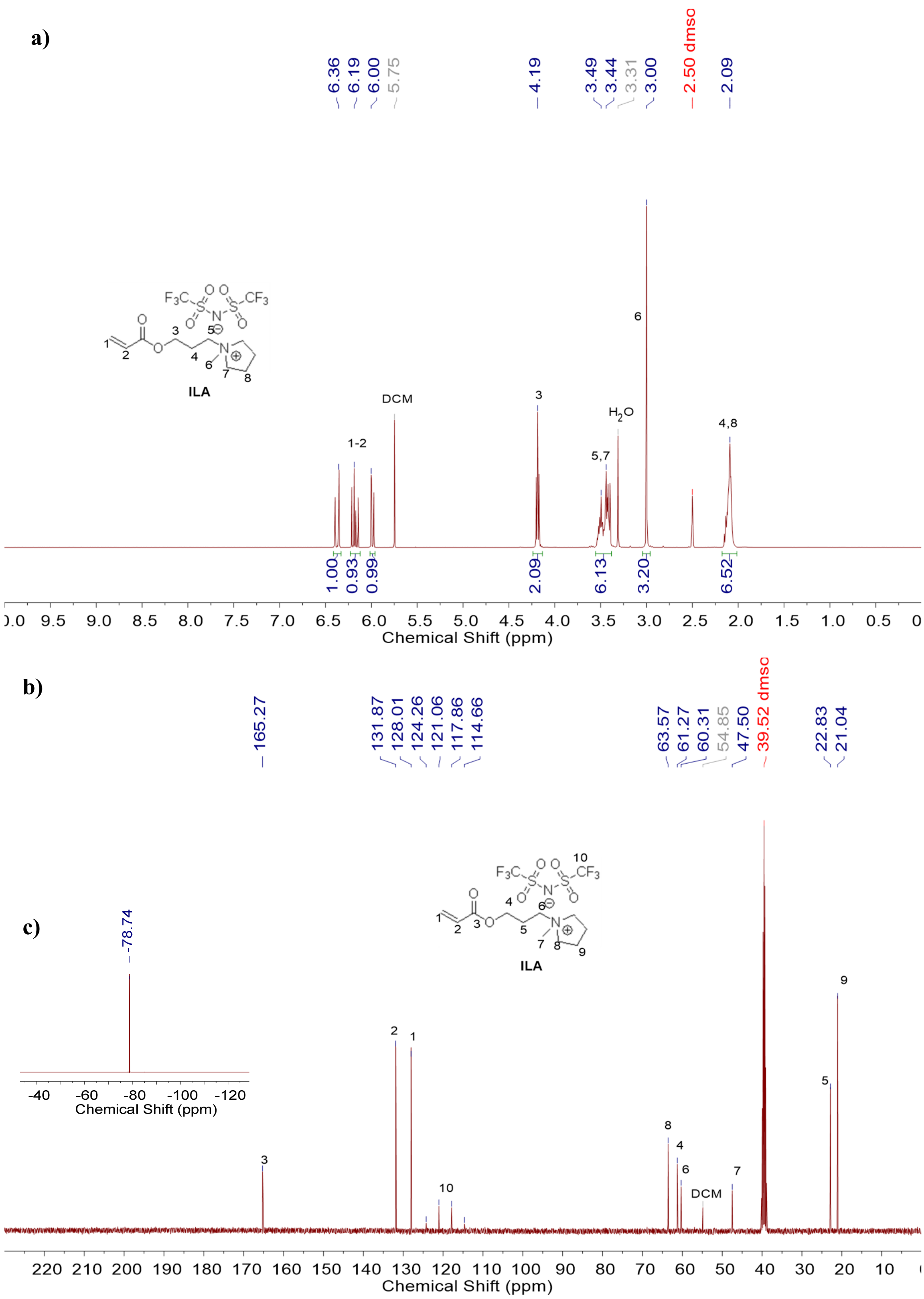

Figure S6 a) ^{1}H, b) ^{13}C, and c) ^{9}F NMR spectra of compound **ILA**.

a)

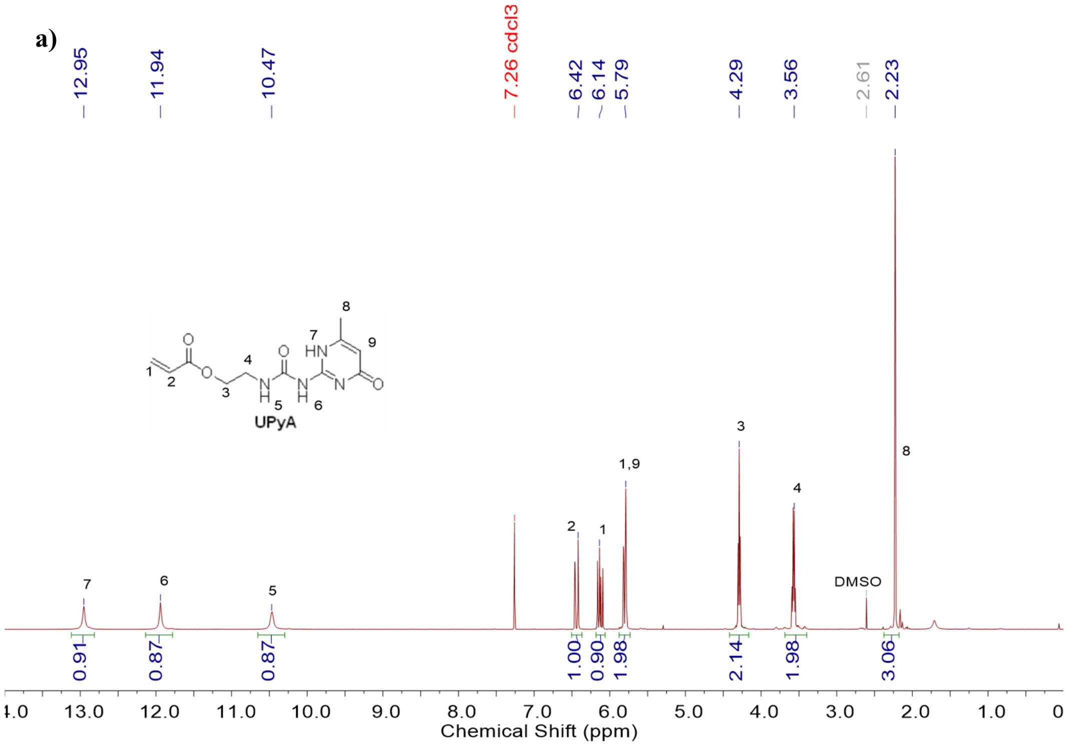

b)

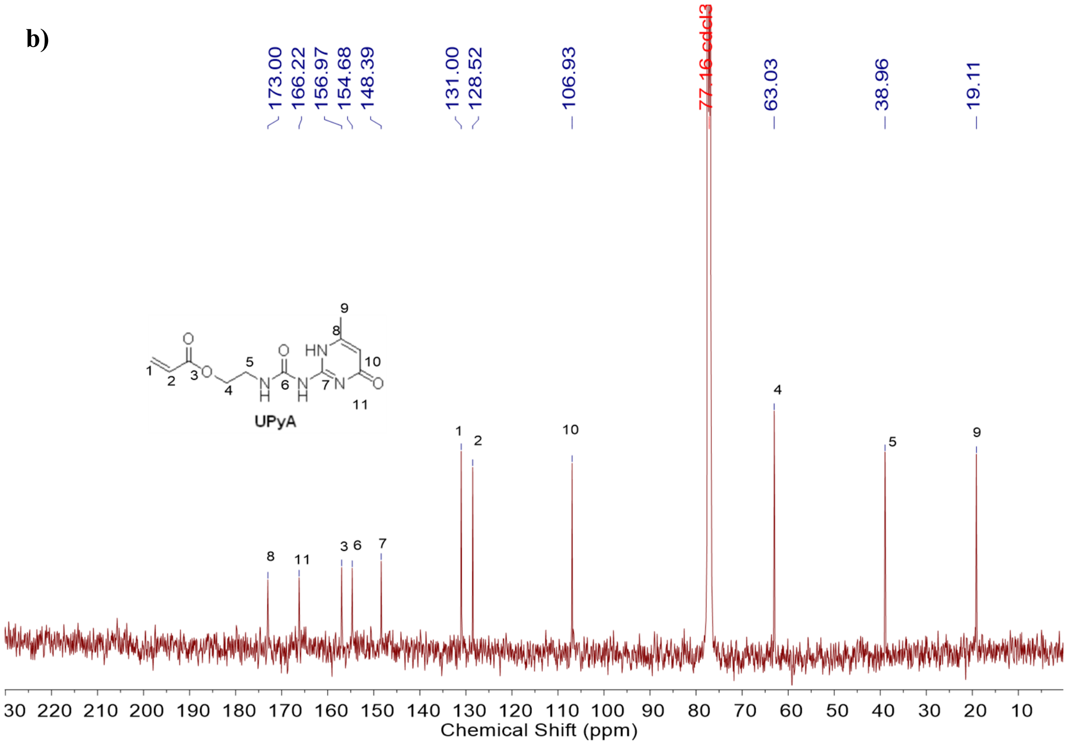

Figure S7 a) ^{1}H and b) ^{13}C NMR spectra of compound **UPyA**.

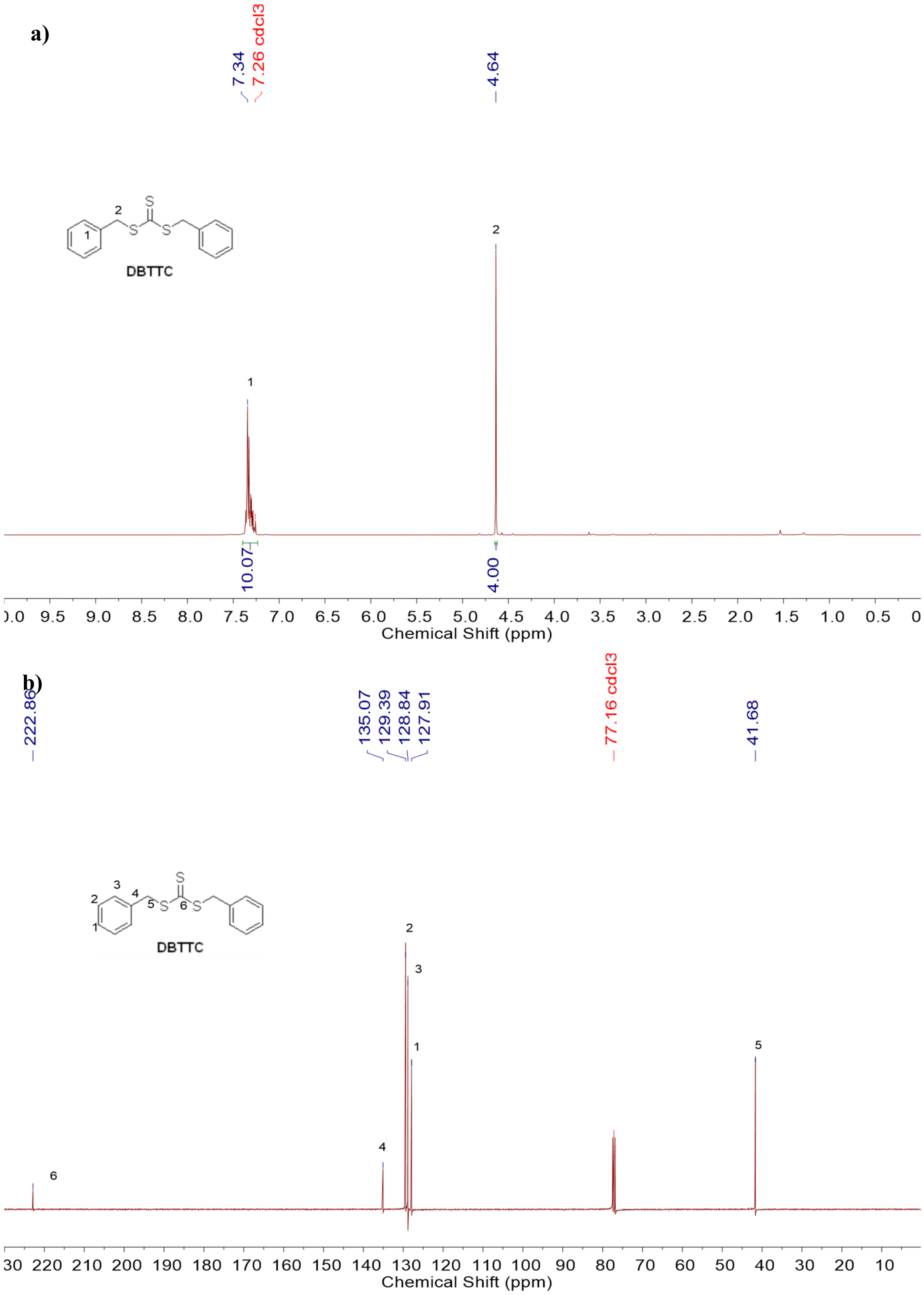

Figure S8 a) ^{1}H and b) ^{13}C NMR spectra of compound **DBTTC**.

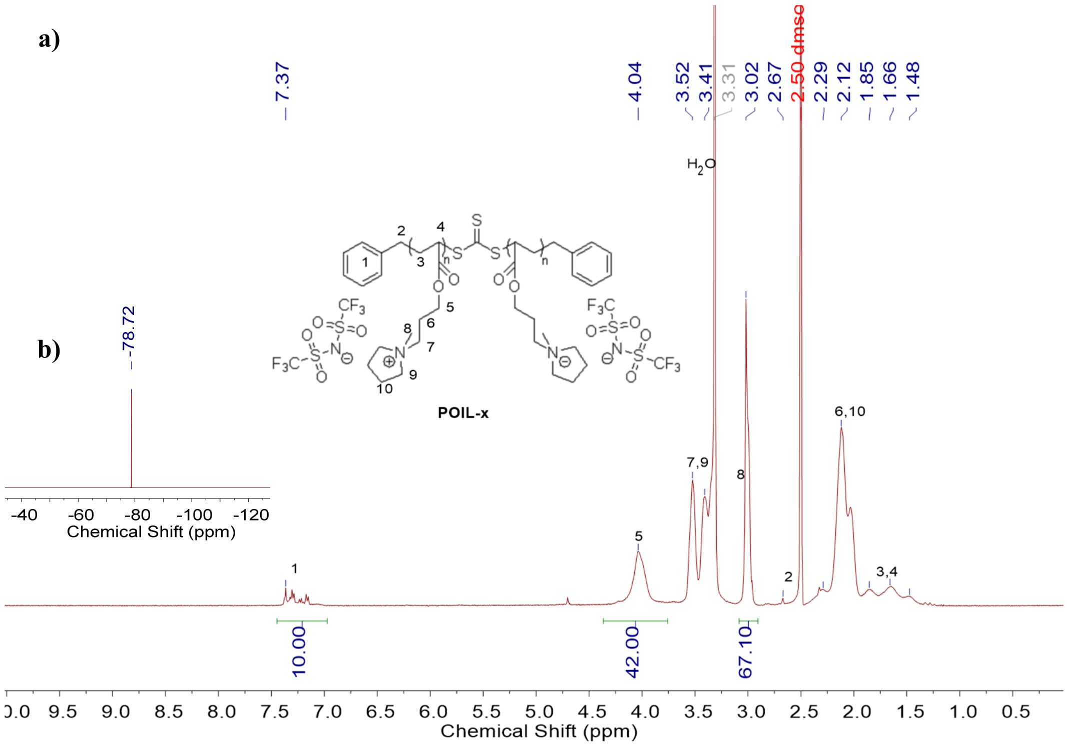

Figure S9 a) ^{1}H and b) ^{9}F NMR spectra of polymer **POIL-x**.

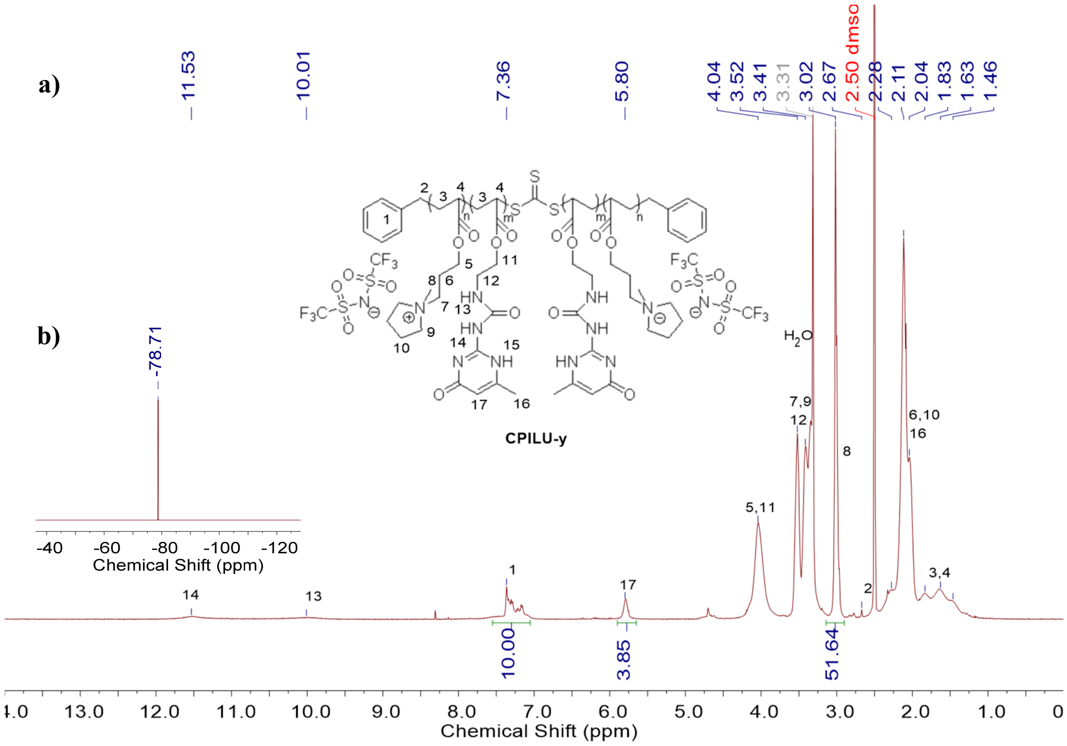

Figure S10 a) ^{1}H and b) ^{9}F NMR spectra of polymer **CPILU-y**.

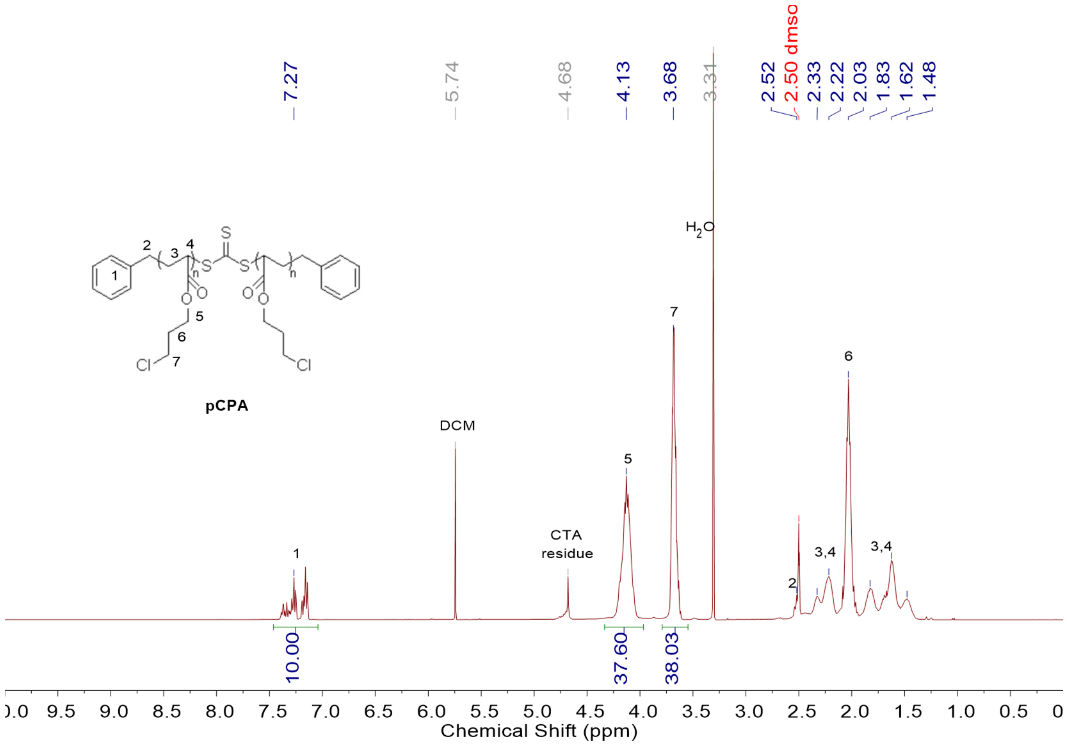

Figure S11 ^{1}H NMR spectra of polymer **pCPA**.

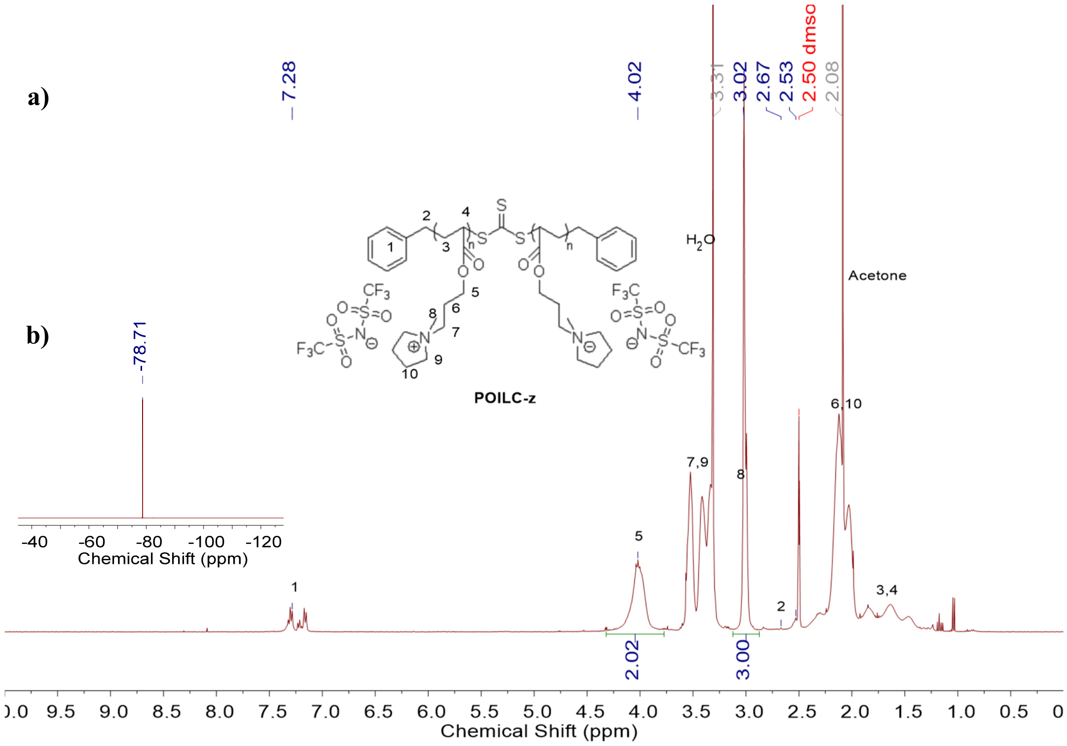

Figure S12 a) ^{1}H and b) ^{9}F NMR spectra of polymer **POILC-z**.

5. Reference

1. Chen, S.; Funtan, A.; Gao, F.; Cui, B.; Meister, A.; Parkin, S.S.P.; Binder, W.H. Synthesis and Morphology of Semifluorinated Polymeric Ionic Liquids. *Macromolecules* **2018**, *51*, 8620-8628, doi:10.1021/acs.macromol.8b01624.
2. Yamauchi, K.; Lizotte, J.R.; Long, T.E. Thermoreversible Poly(alkyl acrylates) Consisting of Self-Complementary Multiple Hydrogen Bonding. *Macromolecules* **2003**, *36*, 1083-1088, doi:10.1021/ma0212801.
3. Aoyagi, N.; Endo, T. Functional RAFT agents for radical-controlled polymerization: Quantitative synthesis of trithiocarbonates containing functional groups as RAFT agents using equivalent amount of CS2. *Journal of Polymer Science Part A: Polymer Chemistry* **2009**, *47*, 3702-3709, doi:10.1002/pola.23410.
4. Cao, W.; Tan, L.; Wang, H.; Yuan, J. Dual-Cationic Poly(ionic liquid)s Carrying 1,2,4-Triazolium and Imidazolium Moieties: Synthesis and Formation of a Single-Component Porous Membrane. *ACS Macro Letters* **2021**, *10*, 161-166, doi:10.1021/acsmacrolett.0c00784.
5. Choi, J.-H.; Ye, Y.; Elabd, Y.A.; Winey, K.I. Network Structure and Strong Microphase Separation for High Ion Conductivity in Polymerized Ionic Liquid Block Copolymers. *Macromolecules* **2013**, *46*, 5290-5300, doi:10.1021/ma400562a.
6. Meek, K.M.; Elabd, Y.A. Alkaline Chemical Stability of Polymerized Ionic Liquids with Various Cations. *Macromolecules* **2015**, *48*, 7071-7084, doi:10.1021/acs.macromol.5b01223.
